# Assessment of Palatal Plane and Occlusal Plane for Determining Anteroposterior Jaw Relation

**DOI:** 10.31729/jnma.4052

**Published:** 2019-02-28

**Authors:** Ujjwal Pyakure, Kamal Babu Thapaliya, Khushboo Singh, Alka Gupta, Sujaya Gupta, Manish Bajracharya, Rabindra Man Shrestha, Praveen Mishra

**Affiliations:** 1 Department of Orthodontics, Kantipur Dental College, Kathmandu, Nepal; 2 Thapaliya Dental Clinic, Chitwan, Nepal; 3 Shangrila Dental Clinic, Kathmandu, Nepal; 4 Department of Periodontics, Kantipur Dental College, Kathmandu, Nepal; 5 Orthodontics Unit, Dental Department, National Academy of Medical Sciences, Bir Hospital, Kathmandu, Nepal

**Keywords:** *ANB angle*, *Nepal*, *palatal plane*, *Wits appraisal*

## Abstract

**Introduction:**

Sagittal jaw relationship is an important parameter for orthodontic treatment planning. Angular and linear measurements both have been proposed and used in orthodontic cephalometrics to assess the sagittal jaw relationships. However, angular measurement has been questioned over the years for its reliability as a result of changes in facial height, jaw inclination and the variable positions of Nasion. So, the objective of our study was to assess the linear anteroposterior jaw relation in a sample of Nepali population using occlusal (Wits appraisal) and palatal planes as reference lines.

**Methods:**

A descriptive cross-sectional study was conducted using the lateral cephalogram of 101 individuals visiting the Department of Orthodontics, Kantipur Dental College, Kathmandu, Nepal. Individuals with Class I skeletal relation were selected using convenience sampling method. Radiographs were standardised and traced. Occlusal and palatal planes were drawn that were bisected by the perpendicular lines from Point A and Point B. The linear distances between the intersections were measured to determine sagittal jaw relations.

**Results:**

In Nepali individuals with normal ANB angle (3.05°±2.511°), the sagittal jaw relation with reference to occlusal (Wits appraisal) and palatal planes were found to be 0.203±3.343mm and 3.574±4.074mm respectively.

**Conclusions:**

Various methods has been proposed and used to assess the sagittal jaw relation and each method has its own strength and limitations. So, it is well advised to use additional cephalometric analysis whenever possible before arriving at any diagnosis and treatment plans.

## INTRODUCTION

Lateral cephalometry is essential for orthodontic diagnosis and treatment planning. Among its applications, the anteroposterior position of jaws relative to each other is extremely important in determining the type of treatment, and in deciding whether orthodontic treatment alone or in combination with surgery is needed.^1^

The most accepted method to determine sagittal jaw relation is “ANB Angle“. However, the reliability and applicability of this method has been questioned.^2,6^ Palatal and occlusal planes as reference lines give more accurate jaw relations of close proximities and exclude the variations commonly encountered with ANB measurements of the craniofacial structure.

The objective of this study was to assess linear anteroposterior jaw relation using occlusal (Wits appraisal) and palatal planes. As per the literature search, no such study has been conducted among Nepali population. Thus this study aims to provide basic guidelines for its use in Nepali individuals.

## METHODS

This descriptive cross-sectional study was conducted in the Department of Orthodontics, Kantipur Dental College during November to December 2018 after obtaining the ethical clearance from the Institutional Review Committee (KDC-IRC). The criteria for inclusion were male and female patients visiting the department fulfilling the following criteria: I.Nepali individuals with Class I skeletal relation (ANB = 2°±2°), 2.Standardised cephalometric technique, 3.Aesthetically pleasing profile. The exclusion criteria were: 1.Unacceptable quality of radiographs, 2.Congenitally missing and impacted teeth, 3.Craniofacial anomalies, 4.Previous orthodontic/orthognathic treatment.

A sample of 101 individuals was calculated and samples were collected using convenience sampling method.


$$\begin{array}{l} \text{n}={\underline{\text{Z}}}^{\underline{2}}\underline{\text{Xp}\left(1-\text{p}\right)}=100.0352\\ {\text{e}}^{2} \end{array}$$

Where,

n = required sample size
Z = 1.96 at 95% confidence level
p = expected outcome proportion; percentage expressed as decimal = 0.93 (93%)^7^
e = margin of error = 0.05 (5%)

Lateral cephalometric radiographs of each individual were taken in natural head position.^8^ Radiographs were traced by a single investigator to mark Point A, Point B, Nasion (N), Anterior Nasal Spine (ANS) and Posterior Nasal Spine (PNS). The functional occlusal plane was drawn by bisecting the molars and premolars, and the palatal plane was drawn by joining ANS and PNS. Perpendicular lines were drawn from point A and point B to the occlusal (AO and BO) and palatal planes (APP and BPP).

AO and BO are the points of contact in the occlusal plane, whereas APP and BPP are the points of contact on the palatal plane. The distances between the intersections were measured. The distance between points AO and BO is known as the Wits appraisal.^9^ The linear distance between APP and BPP was termed “LPP” for the study purpose and was used for the evaluation of anteroposterior jaw relation on the palatal plane. In addition to Wits appraisal and LPP, following angular measurements were taken: SNA, SNB and ANB. Data were entered into SPSS version 17.0, any missing data and outliers were excluded from the analysis. The numerical data were presented as mean, standard deviation and standard error of mean whereas the categorical data were presented as frequency and percentage.

## RESULTS

Wits appraisal and the LPP were found to be 0.203±3.343mm and 3.574±4.074mm respectively (Table 1). Out of 101 patients, 45 (44.6%) were male and 56 (55.4%) were female ranging from 12 to 34 years. Both the Wits and LPP were found to be slightly higher in male (0.522±3.266 mm and 3.656±3.522 mm respectively) compared to female (-0.054±3.412mm and 3.509±4.5 mm respectively). ANB angle was observed to be slightly greater in female (3.16°±2.318°) compared to male (2.91 °± 2.754°) as depicted in (Table 2). Individuals were also categorised according to age group as per the previous studies^10,11^ on the basis of eruption of permanent teeth (Table 3).

**Table 1.** Measusrement of demographic and cephalometric parameters.

Parameters	Mean±SD	SEM	Minimum	Maximum
Age (years)	19.23±4.505	0.448	12	34
Wits (AO-BO, mm)	0.203±3.343	0.332	-8	8
LPP (APP-BPP, mm)	3.574±4.074	0.405	-5	13
SNA (degree)	81.77±3.852	0.383	70	92
SNB (degree)	78.68±3.487	0.347	69	87
ANB (degree)	3.05±2.511	0.250	-3	8

**Table 2.** Demographic and cephalometric parameters according to sex.

	Parameters	**Male n** (%)	**Female n** (%)
		56 (55.4)	45 (44.6)
Age (years)	Mean±SD	18.38±3.979	19.91 ±4.814
	SEM	0.593	0.643
Wits (AO-BO, mm)	Mean±SD	0.522±3.266	-0.054±3.412
	SEM	0.486	0.456
LPP (APP-BPP, mm)	Mean±SD	3.656±3.522	3.509±4.500
	SEM	0.525	0.601
SNA (degree)	Mean±SD	81.93±4.261	81.64±3.524
	SEM	0.635	0.471
SNB (degree)	Mean±SD	79.04±3.444	78.39±3.525
	SEM	0.513	0.471
ANB (degree)	Mean±SD	2.91 ±2.754	3.16±2.318
	SEM	0.410	0.310

**Table 3.** Demographic and cephalometric parameters according to age categories.

Variables		<14 years n (%)	15-25 years n (%)	**>26 years n (%)**
**Gender**	Male	5 (5)	37 (36.6)	3 (3)
	Female	7 (6.9)	42 (41.6)	7 (6.9)
	Total	12 (11.9)	79 (78.2)	10 (9.9)
Wits (AO-BO, mm)	Mean±SD	-0.833±3.121	0.380±3.331	0.050±3.796
	SEM	0.901	0.374	1.20
LPP (APP-BPP, mm)	Mean±SD	4.917±3.175	3.291±4.285	4.20±3.075
	SEM	0.916	0.482	0.972
SNA (degree)	Mean±SD	82.67±2.498	81.52±4.003	82.70±3.974
	SEM	0.721	0.450	1.257
SNB (degree)	Mean±SD	78.50±2.908	78.72±3.541	78.60±4.006
	SEM	0.839	0.398	1.267
ANB (degree)	Mean±SD	4.17±2.406	2.75±2.549	4.10±1.729
	SEM	0.694	0.287	0.547

## DISCUSSION

Lateral cephalometry has been used for the assessment of sagittal jaw relation since it's introduction in 1934 by Hofrathin in Germany and Broadbent in United States of America respectively.^12^ Among various methods used over the decades, assessment using ANB angle remains the most common.^2–4,13^ Freeman (1950)^5^ and Jenkins (1955)^6^ investigated the shortcomings of ANB angle for the first time indicating that the variable positions of Nasion can influence the ANB angle. Furthermore, ANB angle is influenced by jaw rotation and patient's skeletal growth pattern.^2–4^ So to resolve the problem, Jenkins^6^ and Harvold^14^ used functional occlusal plane to evaluate the anteroposterior jaw relation. Finally, when Jacobson presented the Wits appraisal as a better alternative to ANB angle, it eliminated the influence of anatomic variations in Nasion.^9,15,16^

Palatal plane is another skeletal landmark and being closer to the area being surveyed, it is believed by some authors to be the most reliable plane.^17,18^ Furthermore, as demonstrated by various investigators, palatal plane is stable and maintains a constant angular relationship with the anterior cranial base throughout life.^18–21^ Therefore, in the current study occlusal and palatal planes were used to determine the anteroposterior jaw relations in the patients having Class I skeletal profiles as determined by the ANB angle.

The mean value for Wits appraisal in the present study was found to be 0.203±3.343mm which is in agreement with the study of Roth^22^ where Wits appraisal was found to be 0.27±2.34mm in the sample ranging 10-14 years old. Similarly, Wits appraisal according to gender was found to be 0.522±3.266mm for males and -0.054±3.412mm for females which disagrees with the findings of Jacobson (1.17±1.9mm for males and -0.10±1.77mm for females).^9^ The reason for different values could be that the classic study of Jacobson was done in Caucasian population whereas the current study has been done in Nepali population. However, this study doesn't report the findings according race or ethnicity. Age categories (<14, 15-25 and > 26 years) appeared to have no significant influence on Wits appraisal which is in agreement with the findings of Bisharaet al.^23^ This finding suggests that the Wits appraisal is stable and reliable method to assess sagittal jaw relation in every age group.

The normal value for the LPP was determined to be 3.574±4.074mm. This is comparable with the finding by Italia et al.^17^ where 3.62±2.64mm was found to be the anteroposterior jaw relation when palatal plane was used in patients with Skeletal and dental Class I occlusion with normal Wits appraisal value. The LPP values for male (3.656±3.522mm) and female (3.509±4.500mm) is in contrast with Soliman et al.^24^ The authors reported the sagittal jaw relation to be 0.64±0.49mm for male and 0.61±0.39mm for female in Egyptian children when palatal plane was used as reference line. In the current study, the LPP appeared to decrease in 15-25 years old (3.291mm) compared to <14 years old (4.917mm) followed by increase in > 26 years old (4.20mm). This suggests that though the LPP values are similar to previous studies, the LPP measurements may not be used as a reliable parameter for age categories. However studies with larger sample size could predict better results.

The mean ANB angle of total sample was 3.05° which agrees with the finding of Jarvinen^25^ where mean ANB angle was found to be 2.80° in untreated orthodontic patients aged 7 to 14 years with Class I occlusion. ANB angle according to gender was found to be 2.91° in male and 3.16° in female which is less than the values reported by Walker and Kowalski^26^ (4.65° in male and 4.34° in female). This difference may be due to the difference in ethnicity between the studies and the small sample size in the present study. The ANB angle appeared to change significantly with age which is in agreement with the findings of Bishara et al.^23^ These findings suggest that the ANB angle has considerable variations among different racial background, ethnicity and age group.

However, due to possible dentofacial asymmetry, missing, impacted or anomalous teeth, or in mixed dentition where large number of permanent teeth are yet to erupt, it is difficult or even impossible to draw patient's occlusal plane. Similarly in cases where palatal plane is too steep, LPP values tend to increase leading to its unreliability. Other limitations include: The study was carried out in a single institute in a small sample size for short duration. Other cephalometric parameters such as Frankfort Horizontal Plane, Mandibular plane and Sella-Nasion (SN) plane were not considered in the present study.

## CONCLUSIONS

Age categories (<14, 15-25 and >26 years) appeared to have no significant influence on Wits appraisal but ANB angle and LPP changed significantly with age. Thus, from the present study, it is suggested that the sagittal jaw relation in reference to palatal plane (LPP) can be utilised as an adjunctive criteria for proper diagnosis besides the ANB angle and the Wits appraisal. However, among the various methods proposed and used to assess the sagittal jaw relation, each method has its own strength and limitations. So, it would be well advised to use additional cephalometric analysis whenever possible before arriving at any diagnosis and treatment plan.

## Conflict of Interest

**None.**
